# Cephalometric changes during aging in subjects with normal occlusion

**DOI:** 10.1590/1678-7757-2021-0199

**Published:** 2021-10-11

**Authors:** Daniela GARIB, Gabriela Manami NATSUMEDA, Camila MASSARO, Felicia MIRANDA, Rodrigo NAVEDA, Guilherme JANSON

**Affiliations:** 1 Universidade de São Paulo Faculdade de Odontologia de Bauru Departamento de Odontopediatria, Ortodontia e Saúde Coletiva BauruSP Brasil Universidade de São Paulo, Faculdade de Odontologia de Bauru, Departamento de Odontopediatria, Ortodontia e Saúde Coletiva, Bauru, SP, Brasil.

**Keywords:** Age factors, Balanced dental occlusion, Aging, Cephalometry

## Abstract

**Objective:**

To assess craniofacial changes from early adulthood to the seventh decade of life in individuals with normal occlusion.

**Methodology:**

The sample comprised lateral cephalograms of 21 subjects with normal occlusion (11 male, 10 female), taken at 17 (T1) and 61 years of age (T2). Anteroposterior and vertical maxillomandibular relationships, and dentoalveolar and soft tissue changes were analyzed. Interphase comparisons were performed using paired t-tests. Differences between sexes, and subgroups with and without tooth loss were evaluated using t-tests (p<0.05).

**Results:**

Maxillary and mandibular anterior displacement, and facial and ramus height increased from T1 to T2. Maxillary molars showed significant mesial angulation. Maxillary and mandibular molars, and mandibular incisors developed vertically during the evaluation period. Soft tissue changes included a decrease of the nasolabial angle, upper and lower lip retrusion, decrease of upper lip thickness and increase of the lower lip and soft chin thickness. Maxillary incisor exposure by the upper lip decreased 3.6 mm in 40 years. Males presented counterclockwise rotation of the mandible, whereas females showed mandibular clockwise rotation and backward displacement of the chin. The group with tooth loss showed a greater increase of the posterior facial height and ramus height.

**Conclusion:**

We observed aging changes in dentoskeletal structures and soft tissue, as well as sexual differences for craniofacial changes during the maturational process. Subjects with multiple tooth losses showed a greater increase in mandibular ramus height.

## Introduction

Life expectancy remarkably increased in the last century. Craniofacial growth and development are continuous processes, and maturational changes can occur during aging.^1,2^ The number of adults who seek orthodontic treatment for functional or esthetic improvement has increased, and understanding the natural changes that occur throughout life in the craniofacial complex is extremely important. Most previous longitudinal studies evaluated changes in the first two decades of life.^3-6^ Previous studies show that growth continues during adulthood,^1,2,7-15^ rather than immediately stopping after puberty.^14^

Behrents^1^(1984) showed that craniofacial growth is a continuous process during human aging. In his detailed study in a nontreated sample from 25 to 83 years of age, men showed forward and downward mandibular displacement, whereas women showed backward mandibular rotation. The soft pogonion became more prominent, especially in men.^1^ The soft tissue glabella continued to move forward, with retrusion of the upper lip.^1^ The author reported only mild changes between 40 and 80 years of age.^1^ A study by Formby, et al.^10^ (1994) demonstrated that facial profiles straightened with age only in men. A previous maturational study until the fifth decade of life showed that men presented anterior rotation of the mandible, whereas women showed posterior mandibular rotation.^15^ A study with untreated subjects from 17 to 57 years of age reported that changes in the soft tissue were more evident than dentoskeletal changes with aging, including a flattening and elongation of the upper lip, and drooping of the nasal tip and columella.^14^ Only one previous cephalometric study evaluated aging in normal occlusion subjects, showing that facial anteroposterior and vertical dimensions increased from 25 to 46 years of age.^2^

Previous cephalometric studies on craniofacial maturational changes have evaluated untreated samples until the fifth decade of life.^10,14,15^ No previous study has evaluated cephalometric maturational changes in a sample of individuals with normal occlusion with a 40-year follow-up period. Therefore, our study aimed to evaluate the dentoskeletal and soft tissue changes in individuals with normal occlusion from 17 to 61 years of age and the influence of sex and permanent tooth losses on craniofacial changes.

## Methodology

**Figure 1:**
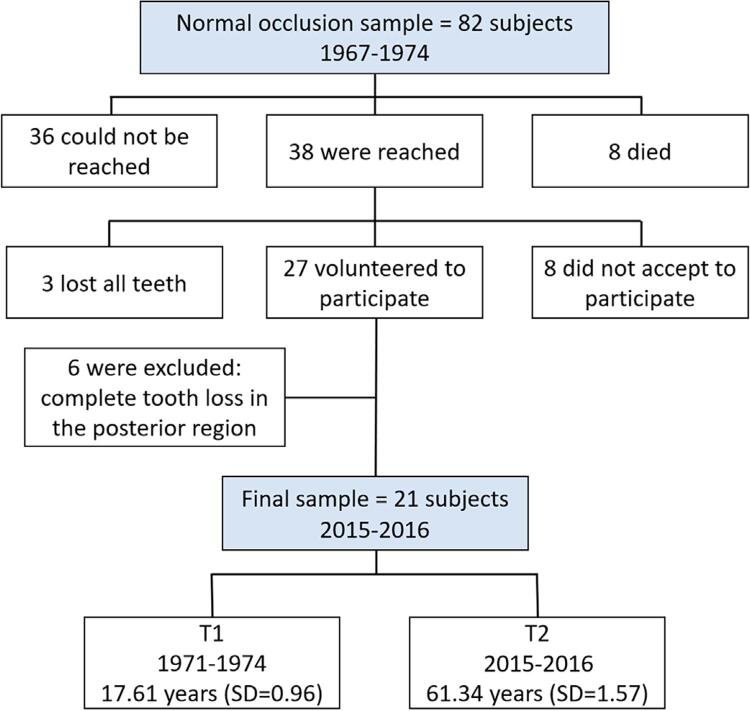
The enrollment process and age distribution during the 40-year follow-up

This observational and longitudinal study was approved by the Ethics in Research Committee of Bauru Dental School, University of São Paulo, protocol #71634917.5.0000.5417. Lateral cephalograms of white Brazilians taken at an initial mean age of 17.61 years (SD=0.96, range: 16.1 to 19.6) were used (T1). All subjects had balanced facial profiles with no excessive protrusion or retrusion and clinically acceptable occlusion in the complete permanent dentition, with dental Class I relationships, normal overjet and overbite, absence of crossbites, maximum 2mm of incisor crowding, and no previous orthodontic treatment. From 2015 to 2016 (T2), the sample was recalled and lateral cephalograms were obtained for this study at a mean age of 61.34 years (SD=1.57, range: 58.6 to 63.6). The exclusion criteria for T2 records were history of orthodontic treatment between T1 and T2, and complete loss of the posterior teeth in one or both dental arches. The final sample consisted of 21 subjects, 11 males with initial age of 17.90 years (SD=0.91) and final age of 61.52 (SD=1.59), and 10 females with initial age of 17.29 years (SD=0.91) and final age of 61.13 years (SD=1.61). The enrollment process is shown in Figure 1. In our sample, up to one tooth loss without prosthetic rehabilitation was observed in nine out of 21 subjects, who were placed in the subgroup without tooth losses. Two or more tooth losses without prosthetic rehabilitation were observed in the other 12 individuals, who were placed in the subgroup with tooth losses. Active periodontal disease was not observed in on clinical examination, considered as bleeding on probing.^17^

All T1 cephalograms were scanned and all cephalograms were analyzed with the Dolphin Imaging^®^ 11.5 software (Dolphin Imaging, Chatsworth, Calif., USA). Correction of 11% and 10% magnification factors for T1 and T2 were performed, respectively. In total, 39 cephalometric variables were evaluated (Table 1). Variables were grouped in Table 1 according to cranial base, maxillary and mandibular skeletal component, maxillomandibular relationship, vertical component, maxillary and mandibular dentoalveolar component, dental relationship, and soft tissue.^14,15^

**Table 1:** Skeletal, dental, soft tissue profile cephalometric variables

Variables	Definition
**Cranial Base**	
S-N (mm)	Distance between S and N points
**Maxillary Skeletal Component**	
SNA (º)	SN to NA angle
CoA (mm)	Condylion to A-point distance
A-NPerp (mm)	A point to nasion-perpendicular
**Mandibular Skeletal Component**	
SNB (º)	SN to NB angle
Co-Gn (mm)	Condylion to Gnathion distance
P-Nperp (mm)	Point P relative to the perpendicular passing through N
**Maxillomandibular relatioship**	
ANB (º)	NA to NB angle
mx/md dif (mm)	Difference between mandibular and maxillary length
**Vertical Component**	
OP.FH (º)	Oclusal plane to Frankfurt plane angle
PP.FH (º)	Palatal plane to Frankfurt plane angle
FMA (º)	Frankfurt mandibular plane
SNGoGn (º)	SN to GoGn angle
UFH (mm)	Distance between N and ANS points
LAFH (mm)	Distance bewteen ANS and Me points
PFH (mm)	Distance between S and Go points
Co-Go (mm)	Condylion to gonion distance
**Maxillary dentoalveolar component**	
Mx1.NA (º)	Maxillary incisor long axis to Na angle
Mx1-NA (mm)	Distance between anterior point of crown of maxillary incisor and NA line
Mx1-PP (mm)	Distance between maxillary incisal edge and palatal plane
Mx6.SN (º)	Angle formed by the long axis of maxillary first molar and SN plane
Mx6-PP (mm)	Mean perpendicular distance between mesial and distal cusps of maxillary first molar and palatal plane
**Mandibular dentoalveolar component**	
Md1.NB (º)	Mandibular incisor long axis to NB angle
Md1-NB (mm)	Distance between the most anterior point of crown of mandibular incisor and NB line
IMPA (º)	Incisor mandibular plane angle
Md1-MP (mm)	Distance between mandibular incisal edge and mandibular plane
Md6.MP (º)	Angle formed by the long axis of mandibular first molar and MP
Md6-MP (mm)	Distance between occlusal point of mandibular first molar and mandibular plane
**Dental relationship**	
Overjet (mm)	Distance between the incisal edge of maxillary and mandibular central incisor, parallel to occlusal plane
Overbite (mm)	Distance between the incisal edge of maxillary and mandibular central incisor, perpendicular to occlusal plane
Mx1.Md1 (º)	Angle between the long axis of Mx1 and Md1
**Soft Tissue Profile**	
Nasolabial Angle (º)	Angle formed between the nose and upper lip
UL cant (º)	Upper lip inclination
UL-E plane (mm)	Distance between upper lip to E plane
LL-E plane (mm)	Distance between lower lip to E plane
UL thickness (mm)	Distance between UL to Mx1
LL thickness (mm)	Distance between LL to Md1
Chin thickness (mm)	Distance between Pog to Pog’
Mx1 exposure (mm)	Mx1 vertical exposition by the upper lip

### Statistical analyses

Mean and standard deviation were estimated for all measurements at T1 and T2. Kolmogorov-Smirnov tests showed normal distribution for all variables. Interphase changes from T1 to T2 were evaluated using paired *t*-tests. Differences between males and females, and between subgroups with and without tooth losses were also investigated with *t*-tests. A 5% significance level was considered. Holm-Bonferroni correction for multiple comparisons was applied.^19^ For the error study, 50% of the sample was randomly remeasured by the same examiner (G.M.N) after a minimum 30-day interval. Random errors were estimated using Dahlberg`s formula^18^, and systematic errors were estimated with dependent *t*-tests, at a 5% significance level. Statistical analyses were performed using the Statistica^©^ software (Statistica for Windows, StatSoft Inc., Tulsa, USA). A post-hoc power analysis was also evaluated using the bilateral parametric test from the GPower software (Version 3.1.9.7, Heinrich-Heine-University, Düsseldorf, Germany).

## Results

Random errors ranged from 0.21mm to 1.82mm for linear variables (overjet and Co-Go, respectively), and from 0.32° to 1.61° for the angular measurements (SNB and Mx1.Md1, respectively). We found no significant systematic error. The achieved power was 0.99, considering a mean change of 6mm in the CoGn variable and a 5% significance level.

### Interphase changes

**Figure 2:**
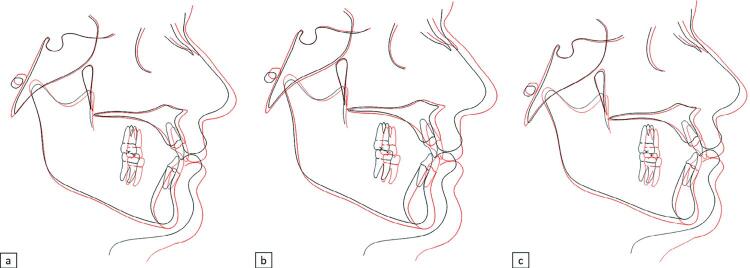
Facial and regional tracing superimposed in the cranial base, centered at S (T1-black; T2-red). a – Average of complete sample. b – Average of male subjects; c - Average of female subjects

From 17 to 61 years of age, we observed a significant increase of 2.74 mm (p<0.001) in the anterior cranial base. The maxillary and mandibular lengths increased 5.40 mm and 6.60mm (p<0.001), respectively. We also observed maxillary and mandibular anterior displacements of 1.47 mm (p<0.001) and 2.42 mm (p<0.001), respectively (Figure 2a, Table 2). The measurements showed a significant increase of the upper and lower facial height (0.74 mm and 2.44 mm, respectively), of the posterior facial height (3.38 mm), and of the ramus height (5.36 mm). Maxillary molars showed a significant mesial angulation of 3.50° (p<0.001), and a vertical development of 2.74 mm (p<0.001). Mandibular incisors and molars also showed a significant vertical development of 1.20 mm and 0.91 mm, respectively. The nasolabial angle significantly decreased (6.10º), and we observed a retrusion of the upper (3.5 mm) and lower lips (2.35 mm). Soft tissue thickness significantly decreased in the upper lip (2.52 mm; p<0.001), whereas the lower lip and soft-tissue chin thickness significantly increased by 1.28 mm (p<0.005) and 2.76 mm (p<0.001), respectively. Exposure of the maxillary incisor decreased 3.68 mm (p<0.001) within 40 years.

**Table 2:** Interphase comparisons in all 21 subjects (paired t-tests)

Variables	T1		T2		T2-T1		CI 95%	p
	Mean	SD	Mean	SD	Mean	SD		
**Cranial Base**								
S-N (mm)	67.34	3.99	70.08	4.05	2.74	0.99	-3.19 to -2.29	<0.001*
**Maxillary Skeletal Component**								
SNA (º)	82.56	3.72	82.84	3.88	0.28	1.49	-0.96 to 0.39	0.398
CoA (mm)	82.56	5.00	87.97	4.97	5.40	1.40	-6.04 to -4.76	<0.001*
A-Nperp (mm)	-0.03	2.86	1.43	2.96	1.47	1.50	-2.15 to -0.78	<0.001*
**Mandibular Skeletal Component**								
SNB (º)	80.16	2.96	80.24	3.46	0.08	1.54	-0.78 to 0.62	0.812
Co-Gn (mm)	114.65	6.63	121.26	7.36	6.60	1.35	-7.22 to -5.98	<0.001*
P-Nperp (mm)	-2.55	3.22	-0.13	4.69	2.42	4.17	-4.32 to -0.52	0.015*
**Maxillomandibular relationship**								
ANB (º)	2.38	1.99	2.63	2.50	0.25	1.42	-0.90 to 0.39	0.426
mx/md dif. (mm)	32.16	3.54	33.28	4.07	1.12	1.73	-1.91 to -0.33	0.007
**Vertical Component**								
OP.FH (º)	7.34	2.37	3.83	4.58	-3.50	4.81	0.94 to 6.06	0.010
PP.FH (º)	-0.08	2.33	-1.37	3.30	-1.28	2.14	0.31 to 2.26	0.012
FMA (º)	24.57	2.83	22.19	4.00	-2.37	3.09	0.96 to 3.78	0.002
SNGoGn (º)	28.76	3.26	28.27	4.65	-0.50	2.40	-0.60 to 1.59	0.357
UFH (mm)	50.56	2.33	51.31	2.47	0.74	0.77	-1.10 to -0.39	<0.001*
LAFH (mm)	65.89	5.83	68.33	6.09	2.44	1.97	-3.34 to -1.54	<0.001*
PFH (mm)	80.76	6.26	84.15	7.22	3.38	2.21	-4.39 to -2.37	<0.001*
Co-Go (mm)	60.20	4.73	65.56	6.14	5.36	2.79	-6.63 to -4.90	<0.001*
**Maxillary dentoalveolar component**								
Mx1.NA (º)	18.80	4.96	21.71	6.73	2.90	6.47	-5.85 to 0.04	0.053
Mx1-NA (mm)	3.43	1.64	3.35	2.66	-0.07	1.88	-0.77 to 0.93	0.190
Mx1-PP (mm)	27.85	3.05	28.59	4.06	0.73	1.66	-1.49 to 0.01	0.055
Mx6.SN (º)	74.44	3.49	77.94	4.62	3.50	4.88	-6.10 to -0.85	0.011*
Mx6-PP (mm)	19.59	2.66	22.34	3.01	2.74	1.86	-3.75 to -1.75	<0.001*
**Mandibular dentoalveolar component**								
Md1.NB (º)	23.09	4.23	24.67	5.27	1.58	4.13	-3.76 to 0.29	0.094
Md1-NB (mm)	4.19	1.62	4.57	1.78	0.37	1.02	-0.84 to 0.08	0.105
IMPA (º)	88.20	4.78	90.06	5.92	1.86	4.54	-3.92 to 0.20	0.074
Md1-MP (mm)	38.40	3.00	39.60	3.06	1.20	1.35	-1.82 to -0.58	<0.001*
Md6.MP (º)	82.04	4.05	80.84	7.24	-1.19	8.19	-2.75 to 5.14	0.533
Md6-MP (mm)	31.61	2.94	32.52	3.45	0.91	1.44	-1.60 to -0.21	0.034*
**Dental Relationship**								
Overjet (mm)	2.49	0.86	2.32	1.43	-0.17	0.95	-0.33 to 0.68	0.481
Overbite (mm)	1.90	1.21	1.76	1.20	-0.13	1.26	-0.53 to 0.81	0.665
Mx1.Md1 (º)	136.52	6.89	132.89	10.88	-3.63	9.27	-0.58 to 7.85	0.087
**Soft tissue**								
Nasolabial Angle (º)	109.45	8.66	103.34	11.06	-6.10	11.17	1.01 to 11.19	0.021*
UL cant (º)	5.51	6.60	5.04	8.59	-0.46	8.24	-3.28 to 4.21	0.797
UL-Eplane (mm)	-3.90	1.85	-7.40	3.30	-3.50	2.37	2.41 to 4.58	<0.001*
LL-Eplane (mm)	-2.49	1.68	-4.84	2.77	-2.35	1.64	1.61 to 3.10	<0.001*
UL thickness (mm)	13.36	1.81	10.84	1.81	-2.52	1.69	1.74 to 3.29	<0.001*
LL thickness (mm)	11.92	1.86	13.21	2.54	1.28	1.91	-2.15 to -0.41	0.005*
Chin thickness (mm)	11.66	1.62	14.42	2.37	2.76	1.86	-3.61 to -1.91	<0.001*
Mx1 exposure (mm)	3.10	1.25	-0.57	2.05	-3.68	1.95	2.79 to 4.57	<0.001*

*Statistically significant after applying Holm-Bonferroni correction (p-values regarded stepwise from 0.002 to 0.05).

### Sexual differences

Long-term craniofacial changes presented sexual dimorphism (Figures 2b, c; Table 3). In males, angular measurements showed slight maxillary and mandibular protrusion (0.89° and 0.93°, respectively), greater increase of mandibular effective length, counterclockwise rotation of the mandible, and greater increase of the ramus height compared to females (Figure 2b). In females, angular measurements showed slight maxillary retrusion (0.38°), backward displacement of the chin (0.86°) and mandibular clockwise rotation (Figure 2c). Additionally, males showed significantly greater retrusion of the upper and lower lips, and greater thickness increase of the soft chin.

**Table 3:** Male and female change comparisons (t-tests)

Variables	Male (n=11)		Female (n=10)		CI 95%	p
	Mean	SD	Mean	SD		
**Cranial Base**						
S-N (mm)	2.78	1.11	2.69	0.90	-0.81 to 1.04	0.840
**Maxillary Skeletal Component**						
SNA (º)	0.89	1.16	-0.38	1.59	0.00 to 2.54	0.049*
CoA (mm)	5.54	1.53	5.25	1.30	-1.02 to 1.59	0.651
A-Nperp (mm)	1.81	1.60	1.09	1.35	-0.63 to 2.09	0.278
**Mandibular Skeletal Component**						
SNB (º)	0.93	1.16	-0.86	1.38	0.63 to 2.96	0.004*
Co-Gn (mm)	7.53	1.02	5.57	0.83	1.08 to 2.80	0.001*
P-Nperp (mm)	3.69	3.65	1.01	4.44	-1.05 to 6.35	0.146
**Maxillomandibular relationship**						
ANB (º)	0.00	1.05	0.54	1.76	-1.86 to 0.72	0.391
mx/md dif. (mm)	1.65	1.44	0.54	1.91	-0.42 to 2.65	0.146
**Vertical Component**						
OP.FH (º)	-4.44	5.14	-2.77	4.71	-6.96 to 3.63	0.511
PP.FH (º)	-1.54	2.21	-1.00	2.14	-2.54 to 1.45	0.573
FMA (º)	-3.50	2.41	-1.13	3.40	-5.05 to 0.29	0.079
SNGoGn (º)	-2.05	1.64	1.22	1.90	-4.90 to -1.65	<0.001*
UFH (mm)	0.53	0.75	0.98	0.76	-1.14 to 0.25	0.197
LAFH (mm)	2.18	1.83	2.73	2.17	-2.38 to 1.29	0.537
PFH (mm)	4.28	2.51	2.40	1.37	1.04 to 5.32	0.050
Co-Go (mm)	6.88	2.53	3.69	2.08	-5.09 to 3.76	0.005*
**Maxillary dentoalveolar component**						
Mx1.NA (º)	3.40	6.23	2.35	7.01	-5.00 to 7.11	0.718
Mx1-NA (mm)	-0.15	1.82	0.00	2.03	-1.91 to 1.62	0.858
Mx1-PP (mm)	0.49	1.55	0.99	1.81	-2.04 to 1.04	0.504
Mx6.SN (º)	4.90	6.04	2.41	3.78	-2.79 to 7.77	0.329
Mx6-PP (mm)	2.74	2.06	2.75	1.83	-2.10 to 2.07	0.991
**Mandibular dentoalveolar component**						
Md1.NB (º)	1.72	4.43	1.43	4.00	-3.58 to 4.17	0.874
Md1-NB (mm)	0.20	1.09	0.56	0.96	-1.30 to 0.56	0.434
IMPA (º)	2.94	4.77	0.67	4.18	-1.84 to 0.56	0.261
Md1-MP (mm)	1.02	1.62	1.40	1.04	-1.65 to 0.87	0.541
Md6.MP (º)	0.77	8.87	-3.37	7.23	-3.75 to 12.04	0.283
Md6-MP (mm)	1.05	1.33	0.74	1.63	-1.14 to 1.72	0.657
**Dental Relationship**						
Overjet (mm)	-0.65	0.83	0.20	0.90	-1.82 to 0.09	0.072
Overbite (mm)	-0.43	0.89	0.09	1.49	-1.94 to 0.84	0.427
Mx1.Md1 (º)	-4.84	8.74	-2.30	10.12	-11.16 to 6.07	0.543
**Soft tissue**						
Nasolabial Angle (º)	-2.57	8.54	-9.99	12.82	-2.44 to 17.28	0.132
UL cant (º)	-3.76	6.60	3.16	8.63	-13.90 to 0.05	0.517
UL-Eplane (mm)	-5.14	1.81	-1.69	1.40	-4.93 to -1.93	<0.001*
LL-Eplane (mm)	-3.19	1.73	-1.43	0.92	-3.01 to -0.45	0.010*
UL thickness (mm)	-3.04	1.62	-1.94	1.66	-2.58 to 0.43	0.143
LL thickness (mm)	1.60	2.05	0.94	1.77	-1.12 to 2.42	0.447
Chin thickness (mm)	3.87	1.31	1.55	1.64	0.97 to 3.68	0.002*
Mx1 exposure (mm)	-4.19	1.60	-3.12	2.22	-2.80 to 0.72	0.219

*Statistically significant after applying Holm-Bonferroni correction (p-values regarded stepwise from 0.006 to 0.05).

### Influence of tooth losses

The subgroup with tooth loss showed a greater increase of the posterior facial height (4.25 mm; p<0.035) and ramus height (7.01 mm; p<0.001) compared to the group without multiple tooth loss (Table 4).

**Table 4:** Comparison between subgroups with and without tooth loss (t-tests)

Variables	Without tooth loss (n=9)		With tooth losses (n=12)		CI 95%	p
	Mean	SD	Mean	SD		
**Cranial Base**						
S-N (mm)	2.56	0.98	2.88	1.03	-1.25 to 0.61	0.480
**Maxillary Skeletal Component**						
SNA (º)	0.52	1.02	0.10	1.79	-0.99 to 1.81	0.544
CoA (mm)	5.29	1.24	5.49	1.55	-1.52 to -0.15	0.755
A-Nperp (mm)	1.32	0.62	1.58	1.94	-1.67 to 1.15	0.705
**Mandibular Skeletal Component**						
SNB (º)	-0.11	1.06	0.22	1.86	-1.79 to 1.11	0.634
Co-Gn (mm)	5.95	0.63	7.09	1.56	-2.30 to 0.02	0.054
P-Nperp (mm)	1.37	2.18	3.21	5.16	-5.69 to 2.02	0.331
**Maxillomandibular relationship**						
ANB (º)	0.74	1.07	-0.11	1.58	-0.42 to 2.14	0.176
mx/md dif. (mm)	0.66	1.50	1.59	1.49	-2.32 to 0.44	0.171
**Vertical Component**						
OP.FH (º)	-2.47	4.22	-5.92	5.14	-1.56 to 8.46	0.162
PP.FH (º)	-0.73	1.44	-1.70	2.52	-1.00 to 2.94	0.318
FMA (º)	-1.41	1.39	-3.10	3.83	-1.13 to 4.50	0.225
SNGoGn (º)	0.13	1.47	-0.96	2.89	-1.11 to 3.31	0.312
UFH (mm)	0.64	0.78	0.82	0.78	-0.90 to 0.54	0.609
LAFH (mm)	2.10	0.78	2.69	2.54	-2.43 to 1.26	0.513
PFH (mm)	2.23	1.09	4.25	2.48	-3.88 to -0.15	0.035*
Co-Go (mm)	3.16	1.83	7.01	2.20	-5.74 to -1.95	<0.001*
**Maxillary dentoalveolar component**						
Mx1.NA (º)	2.71	6.42	3.05	6.79	-6.46 to 5.79	0.909
Mx1-NA (mm)	-0.74	1.26	0.42	2.15	-2.85 to 0.52	0.166
Mx1-PP (mm)	0.80	1.20	0.68	1.98	-1.44 to 1.69	0.865
Mx6.SN (º)	4.43	5.27	2.30	4.42	-3.19 to 7.46	0.405
Mx6-PP (mm)	2.50	1.33	3.05	2.48	-2.61 to 1.51	0.577
**Mandibular dentoalveolar component**						
Md1.NB (º)	0.70	4.43	2.25	3.95	-5.39 to 2.29	0.408
Md1-NB (mm)	0.07	1.12	0.60	0.92	-1.46 to 0.39	0.244
IMPA (º)	1.42	4.29	5.06	5.37	-8.21 to 0.92	0.111
Md1-MP (mm)	1.09	0.97	1.28	1.62	-1.47 to 1.08	0.757
Md6.MP (º)	-4.71	6.40	1.97	8.62	-14.11 to 0.74	0.074
Md6-MP (mm)	1.05	1.63	0.92	1.34	-1.47 to 1.41	0.969
**Dental Relationship**						
Overjet (mm)	0.22	0.96	-0.67	0.72	-0.03 to 1.83	0.058
Overbite (mm)	-0.13	1.30	-0.14	1.30	-1.40 to 1.42	0.990
Mx1.Md1 (º)	-0.83	6.43	-5.73	10.73	-3.56 to 13.36	0.240
**Soft tissue**						
Nasolabial Angle (º)	-0.34	9.31	-0.55	7.77	-7.58 to 8.01	0.954
UL cant (º)	-3.76	6.60	3.16	8.63	-14.83 to 5.09	0.517
UL-Eplane (mm)	-3.10	2.69	-3.79	2.18	-1.54 to 2.91	0.525
LL-Eplane (mm)	-2.45	1.56	-2.28	1.75	-1.72 to 1.37	0.815
UL thickness (mm)	-2.94	1.46	-2.20	1.85	-2.30 to 0.83	0.340
LL thickness (mm)	0.65	1.54	1.76	2.08	-2.83 to 0.62	0.195
Chin thickness (mm)	1.93	2.02	3.39	1.54	-3.07 to 0.17	0.077
Mx1 exposure (mm)	-3.45	2.20	-3.85	1.82	-1.43 to 2.24	0.649

*Statistically significant after applying Holm-Bonferroni correction (p values regarded stepwise from 0.025 to 0.05).

## Discussion

To our knowledge, this is the first cephalometric study evaluating aging up to the seventh decade of life in normal occlusion subjects. Studies have evaluated maturational changes of the craniofacial complex in untreated individuals.^1,2,8,10,14,15,20^ One of the limitations of longitudinal studies is the difficulty in collecting data, which restricts the sample size.^14,15,21,22^ The difficulty in recalling the sample after 47 years were relevant considering the subjects had changed phone numbers and addresses. Additionally, women had adopted marital names. After trying to reach all the 82 subjects from the initial sample group, 24 were reached, of which 21 accepted to participate. A *post-hoc* power analysis showed a statistical power of 99%, validating our results. Behrents’ study from 1984 had only 4 subjects followed after 40 years of age. However, his results represented a very important contribution to clinical orthodontics on adult facial growth, and still are unique. Our study sample was selected from a historical sample of normal occlusion subjects collected in the late 60s.Therefore, a limited number of subjects were available for a second evaluation 40 years later. Despite the small sample, we achieved an adequate power, and the results showed several changes in skeletal and soft tissues with aging, confirming that craniofacial development continued into adulthood, as previously reported^1^ (Figure 2). Soft tissues presented most significant changes compared to those of dentoskeletal tissues, agreeing with previous studies performed in untreated individuals.^1,10,14,15^

Considering the complete sample, cranial base length showed a significant increase over 44 years (Figure 2a, Table 2). This finding agrees with previous studies^14,23^, and is associated to the anterior movement of frontal and nasal bones^1,24^, or in combination with posterior movement of the sella.^24^ Maxillary length and protrusion increased significantly. Mandibular length and protrusion also significantly increased from T1 to T2. These results corroborate previous longitudinal studies after adolescence showing that the maxilla and mandible continue to grow during adulthood.^2,10,14,15,23,25^ Bone apposition on the anterior surface of the symphysis might also have occurred, contributing to the anterior mandibular displacement.^23^

In general, the upper facial height increase might be associated with the downward movement of the anterior nasal spine. The lower anterior facial height increase was greater than the upper facial height increase, and probably subsequent to teeth eruption.^1,14^ The ramus height increase explains most of the posterior facial height increase^1^ (Figure 2a). Our vertical skeletal findings corroborate previous studies on maturational changes in untreated subjects.^1,10,14,15^

Dentoalveolar changes included significant mesial angulation of the maxillary molars. Increase of the maxillary molar angulation can be explained by a mesial shift of posterior teeth throughout life^1,26,27^, and mesiodistal tooth size reduction during aging.^21^ Significant vertical development of the maxillary and mandibular first molars and mandibular incisors were also observed, confirming that teeth continue to erupt over time during adulthood.^5,15^ A study by West and McNamara^15^(1999), with subjects from 17 to 47 years of age, showed vertical developments of 1.1 mm, 1.8 mm and 1.6 mm for maxillary and mandibular first molars and mandibular incisors, respectively. A previous 40-year follow up of subjects with normal occlusion using digital dental models showed increases of clinical crown height, slight incisor crowding, decrease of mesiodistal tooth size, decrease of the mandibular intercanine width and arch perimeter, and, finally, slight overbite reduction during aging.^21^ These changes should be considered during orthodontic treatment planning in adult patients.

Over 40 years, changes to the facial soft tissue were quantitatively more expressive than dentoskeletal changes (Table 2). The nasolabial angle decreased 6.1° despite the retrusion, and decreased thickness of the upper lip, indicating that downward movement of the nasal columella occurred during aging (Figure 2a). A previous study measured the vertical development of the columella relative to the Frankfurt plane, showing an increase of 3.8 mm from 17 to 57 years of age, which confirms a nasal downward movement over time.^14^ Other studies also reported similar findings of downward movement of the nose with ageing.^1,10,15,20,28^ Lips became more retruded, specially the upper lip. Considering the inexistence of significant changes for maxillary and mandibular incisor protrusion with aging, lip retrusion is probably mostly related to forward movement of the nose and chin, and to an actual decrease of upper lip thickness. Similar findings were reported in previous studies in untreated subjects.^1,10,14^ Reduction of upper lip thickness observed in our study might be related to the natural aging process of the skin which becomes less consistent and inelastic over time.^1,10,14,29^ In contrast to the upper lip, the lower lip showed a slight thickness increase of 1.28 mm. Behrents^1^(1984) also found more prominent lower lip during aging, contributing to a deepening of the mentolabial sulcus. Soft chin thickness also increased, corroborating previous studies.^1,14^ A study in normal occlusion subjects between 20 and 30 years of age showed that lower lip and soft chin thickness increased a mean of 0.55mm and 0.51mm, respectively.^30^ Both the nose and soft chin changes should increase the perception of bi-retruded lips with aging. Additionally, maxillary incisor exposure decreased, probably due to the upper lip vertical changes added to the natural force of gravity and to the occurrence of an incisal edge wear in the central incisors.^21^ The 3.68 mm reduction in maxillary incisor exposure over a 40-year follow-up indicates a rate of approximately 1 mm loss of maxillary incisor display every decade. A previous study in Korean subjects between 20 and 69 years of age showed a gradual decrease in maxillary incisor exposure of 4 mm, and a 3 mm increase in mandibular tooth exposure in the rest position.^31^ Other studies found similar changes.^32-34^

Craniofacial changes over the 40-year period presented sexual differences (Figure 2b, c; Table 3). Males presented maxillary and mandibular protrusion, and counterclockwise rotation of the mandible (Figure 2b). In contrast, females showed slight maxillary and mandibular retrusion, and clockwise rotation of the mandible (Figure 2c). Our results agree with previous studies in untreated subjects.^1,14,15^ Pecora, et al.^14^(2008) also showed sexual mandibular growth differences in subjects between 17 and 47 years, among which women showed more vertical changes and posterior rotation of the mandible, whereas men showed a more anterior mandibular rotation. Both sexes showed increase in the mandibular ramus height (Co-Go) from T1 to T2; however, men showed a significantly greater increase than women which corroborate other studies.^1,2,15^ The greater increase in ramus height in men could be associated with the counterclockwise rotation of the mandible.^1^ Furthermore, several men had not completed their circumpubertal active growth at T1 considering the wide age range. Considering the cervical vertebral maturation,^35^ seven out of 11 male subjects were at CS5 and three were still at CS4 at 17 years of age. On the contrary, six out of 10 female subjects showed stage CS6 at T1. These differences in sexual skeletal maturation at T1 could explain the greater changes observed in men between 17 and 61 years of age. The sexual differences in soft tissue changes consisted in greater retrusion of the upper and lower lips in males that might be related to the greater increase of nose and soft chin dimensions. These findings corroborate previous studies.^1,2,15,36^ Soft chin thickness increased more in males than in females in a 3:1 ratio (Figure 2b and c). These results support previous studies showing similar findings.^1,14,29,33^

The subgroup with tooth loss showed greater increase of the posterior facial height and ramus height than the subgroup without multiple tooth loss (Table 4). These results might be due to a counterclockwise rotation of the mandibular plane that occurs in subjects with posterior tooth losses, decreasing the vertical occlusal dimension. Our findings corroborate a previous study that compared mandibular size of edentulous, old dentate and young dentate individuals and showed significant greater ramus length in edentulous individuals, compared to young and old dentate individuals.^37^ Hutchinson, Farella and Kramer (2015) found edentulous mandible with greater ramus height compared to dentate and partially edentulous mandibles. Differences between subgroups should be interpreted with caution due to the reduced sample power – a limitation of our study. Despite this limitation, this is the first cephalometric study following subjects with untreated normal occlusion until 70 years of age.

In short, this study has provided further evidence that the craniofacial complex continues to change from early to mature adulthood, probably due to terminal growth and bone remodeling processes throughout life.^1^ Several changes in the dentoskeletal and soft tissues might be expected with aging in subjects with normal occlusion. As clinical considerations, orthodontists should be very careful when suggesting procedures that reduce lip protrusion, straighten the facial profile, and decrease maxillary incisor display, to avoid accelerating facial aging.

## Conclusions

Between 17 and 61 years of age, normal occlusion subjects present anterior displacement of the maxilla and mandible, and increases in facial heights; maxillary molars showed mesial angulation and extrusion. The mandibular incisors and molars also extruded with aging. They also showed closure of the nasolabial angle, retrusion of the lips, increase of the soft-tissue chin, and reduction of the maxillary incisor exposure occurred during aging. These subjects also showed sexual differences in the craniofacial changes from early to mature adulthood. Finally, subjects with multiple tooth losses presented a greater increase in mandibular ramus height.
